# Marine-Derived Macrocyclic Alkaloids (MDMAs): Chemical and Biological Diversity

**DOI:** 10.3390/md18070368

**Published:** 2020-07-17

**Authors:** Hanan I. Althagbi, Walied M. Alarif, Khalid O. Al-Footy, Ahmed Abdel-Lateff

**Affiliations:** 1 Department of Chemistry, Faculty of Science, University of Jeddah, P.O. Box 13151, Jeddah 21493, Saudi Arabia; halthagbi@uj.edu.sa; 2 Department of Chemistry, Faculty of Science, King Abdulaziz University, P.O. Box 80203, Jeddah 21589, Saudi Arabia; kalfooti@kau.edu.sa; 3 Department of Marine Chemistry, Faculty of Marine Sciences, King Abdulaziz University, P.O. Box 80207, Jeddah 21589, Saudi Arabia; 4 Department of Natural Products and Alternative Medicine, Faculty of Pharmacy, King Abdulaziz University, P.O. Box 80260, Jeddah 21589, Saudi Arabia; aabdellatteff@kau.edu.sa; 5 Department of Pharmacognosy, Faculty of Pharmacy, Minia University, Minia 61519, Egypt

**Keywords:** marine natural products, macrocyclic alkaloids, potential drugs, biological activity

## Abstract

The curiosity and attention that researchers have devoted to alkaloids are due to their bioactivities, structural diversity, and intriguing chemistry. Marine-derived macrocyclic alkaloids (MDMAs) are considered to be a potential source of drugs. Trabectedin, a tetrahydroisoquinoline derivative, has been approved for the treatment of metastatic soft tissue sarcoma and ovarian cancers. MDMAs displayed potent activities that enabled them to be used as anticancer, anti-invasion, antimalarial, antiplasmodial, and antimicrobial. This review presents the reported chemical structures, biological activities, and structure–activity relationships of macrocyclic alkaloids from marine organisms that have been published since their discovery until May 2020. This includes 204 compounds that are categorized under eight subclasses: pyrroles, quinolines, bis-quinolizidines, bis-1-oxaquinolizidines, 3-alkylpiperidines, manzamines, 3-alkyl pyridinium salts, and motuporamines.

## 1. Introduction

The marine environment is one of the harshest atmospheres on the earth due to its diverse ranges of light, temperature, pressure, and nutrient circumstances [1]. These conditions enable marine organisms to produce extremely different and unprecedented metabolites with a wide range of bioactivities [2,3]. The organisms that live in this environment have immense genetic and biochemical diversity that, being the source of unexplored bioactive products, could be beneficial for the development of potential drugs [4].

The discovery of such drugs is expensive, time-consuming, and risky because it is achieved through complicated processes. Moreover, drug discovery is supported by the combination of databases with dereplication methodologies, such as computer-assisted structure elucidation (CASE) and mass spectrometry or nuclear magnetic resonance (NMR) spectroscopy (metabolite- guided and genome-guided approaches) [3].

Twenty marine-derived compounds have been considered in different clinical trial phases, ranging from Phase I to III. Moreover, four macrocyclic compounds out of eight approved marine-derived drugs have been approved by the Food and Drug Administration (FDA), Australia’s Therapeutic Goods Administration, the European Medicines Agency (EMA), and the Japanese Ministry of Health [5].

Marine macrocyclic natural products (MMNPs) include four main subclasses according to their structural differences, namely, cyclic depsipeptides, diterpenes, macrolides, and macrocyclic alkaloids. MMNPs have been reported from different sources, including sponges, algae, fungi, mollusks, cyanobacteria, and gorgonians [6].

The unprecedented skeletons of MMNPs and structural complexity have an important role in the potency of their bioactivities. This has enhanced the discovery of anticancer drugs such as trabectedin [7], which is a tetrahydroisoquinoline alkaloidal derivative that has been approved by the FDA and the European Agency for the Evaluation of Medicinal Products (EMEA) as an anticancer drug. Ingenamine G has been shown to exhibit potent cytotoxic effects against HCT-8 (colon), B16 (leukemia), and MCF-7 (breast) cancer cell lines, as well as antibacterial effects against *Staphylococcus aureus*, *Escherichia coli*, four oxacillin-resistant *S. aureus* strains, and *Mycobacterium tuberculosis* H37Rv [8]. The potent blocking activity of xestospongin A, araguspongine B, demethylxestospongin B, and araguspongines C and D on IP3-mediated Ca^2+^ release from the endoplasmic reticulum vesicles of the rabbit cerebellum has been published [9]. Finally, the antimalarial activity of manzamines has been reported [10].

**Figure 1:**
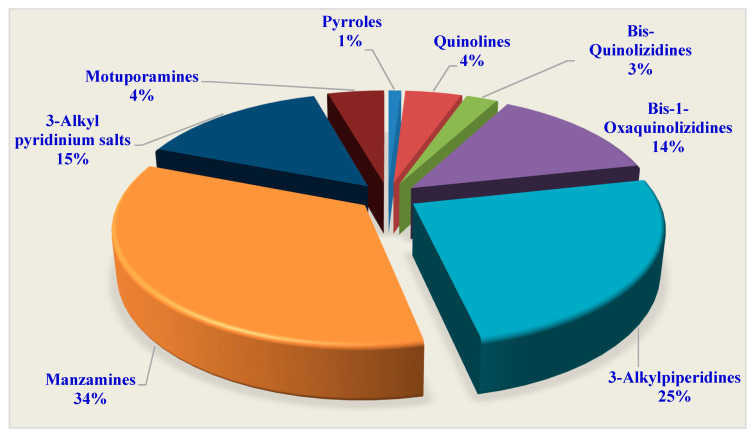
Percentage of marine-derived macrocyclic alkaloids’ subclasses.

This review discusses the reported chemical structures, biological effects, and structure–activity relationships (SARs) of eight subclasses of marine-derived macrocyclic alkaloids-pyrroles, quinolines, bis-quinolizidines, bis-1-oxaquinolizidines, 3-alkylpiperidines, manzamines, 3-alkyl pyridinium salts, and motuporamines. Also included within this review are 204 compounds that have been reported since their discovery until May 2020 (Figure 1 and Table 1). 

## 2. Macrocyclic Alkaloids

### 2.1. Macrocycles Containing a Pyrrole Moiety

#### Densanins

**Figure 2:**
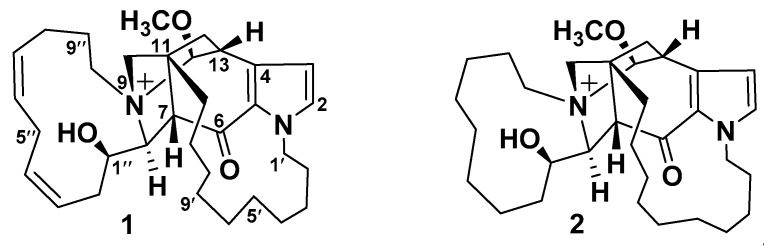
Structures **1** and **2**.

Densanins A (**1**) and B (**2**) were isolated from the sponge *Haliclona densaspicula* [11]. Densanins are fused hexacyclic diamine alkaloids with a pyrrole ring that fused to the tricyclic core (Figure 2). Compounds **1** and **2** displayed potent inhibitory effects against lipopolysaccharide-induced nitric oxide production in BV2 microglial cells, with IC_50_ values of 1.05 and 2.14 μM, respectively [11]. These cells are macrophages of the central nervous system (CNS) and are considered to be a primary form of the active immune defense in the CNS, particularly in Alzheimer’s and Parkinson’s diseases. Microglia are chronically activated and promote the release of cytokines, which further disrupt normal CNS activities. Thus, the inhibitory effect of inflammatory mediator production in these cells can mitigate the effects of inflammation. Therefore, both metabolites could have potential for development of drugs for treatment of neurodegenerative diseases such as Alzheimer’s and Parkinson’s diseases [12].

### 2.2. Macrocycles Containing a Quinoline Moiety 

#### Njaoamines

**Figure 3:**
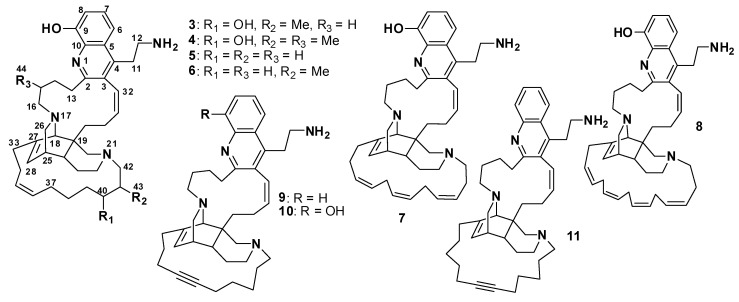
Structures of **3**–**11**.

Njaoamines are a group of biologically active alkaloids containing a tricyclic nitrogenated nucleus with two hydrocarbon bridges, one of which embeds an 8-hydroxyquinoline moiety. Njaoamines A–F (**3–8**) (Figure 3) were isolated from the Haplosclerida sponge *Reniera* sp. [13], whereas njaoamines G (**9**) and H (**10**) were isolated from the marine sponge *Neopetrosia* sp. [14] and njaoamine I (**11**) from the *Haliclona* (*Reniera*) sp. (Figure 3) [15]. Njaoamines showed cytotoxic effects against NSLC A-549 (lung), HT-29 (colon), and MDA-MB-231 (breast) human tumor cell lines. Compounds **3–8** and **11** showed cytotoxic effects, with GI_50_ values ranging from 1.5 to 7.2 μΜ against NSLC A-549, from 1.4 to 6.7 μΜ against HT-29, and from 1.5 to 7.2 μΜ against MDA-MB-23 [13,15]. Compounds **9** and **10** exhibited potent toxicity toward brine shrimp, with LD_50_ values of 0.17 and 0.08 μg/mL, respectively [14]. Compound **11** displayed neither an inhibitory effect on human recombinant topoisomerase 1 nor inhibition of the interaction between programmed cell death protein 1(PD-1) and its natural ligand, programmed death-ligand 1(PD-L1), even at the highest concentration tested, 100 μM [15].

### 2.3. Macrocycles Containing a Bis-Quinolizidine Moiety

#### Petrosins

**Figure 4:**
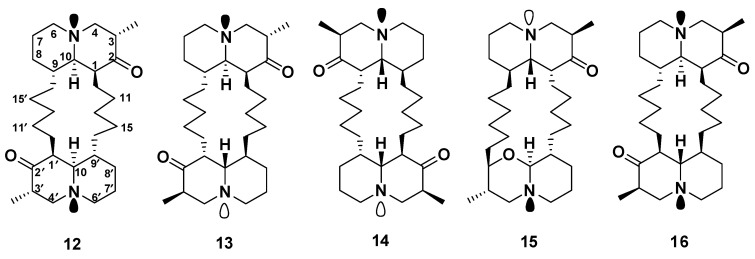
Structures of **12**–**16**.

Petrosin (**12**), the first reported bis-quinolizidine scaffold linked through a C-16 ring from *Petrosia seriata* [16]. Later on, two ichthyotoxic bis-quinolizidine alkaloids, petrosins A (**13**) and B (**14**), were isolated from the same sponge [17]. In 1988, the structure of petrosin A (13) was revised through 2D-NMR studies by Braekman et al. [18]. Aragupetrosine A (**15**), along with **12** and **13,** was reported from an Okinawan marine sponge, *Xestospongia* sp. [19] (Figure 4). Compound **15** consists of the 3β-methyl-*trans*-2-oxaquinolizidine and 3‘α-methyl-*trans*-1-oxoquinolizidine moieties joined by two alkyl chains, which can be viewed as one half moiety of petrosin (**12**) and the 3` α-methyl-*trans*-1-oxoquinolizidine group [19].

Compounds **12** and **13**, isolated from *Xestospongia muta*, did not show growth inhibition against LU-1 (lung), HepG-2 (liver), HL-60 (leukemia), MCF-7 (breast), and SK-Mel-2 (melanoma) human cancer cells [20]. However, compounds **12**, **13**, and **15** exhibited vasodilative activity, and **12** and **13** were two-fold more active than papaverine [19]. In addition to ichthyotoxic and vasodilative activities, **12** and **13**, isolated from the sponge *P. similis,* showed significant in vitro antiviral activity against human immunodeficiency virus (HIV-1), with IC_50_ values of 41.3 and 52.9 μM, respectively [21]. Moreover, **12** and **13** inhibited the early replication of HIV-1 as indicated by multinuclear activation of a galactosidase indicator (MAGI) assay, with giant cell formation and inhibition of human immunodeficiency virus-1 reverse transcriptase (RT) at 10.6 and 14.8 μM [21], respectively. Interestingly, **12** did not only show higher activity against HIV than **13** but is also more stable than **13** [21]. Xestosin A (**16**), another bis-quinolizidine-containing macrocycle, was isolated from the Papua New Guinean sponge *Xestospongia exigua* [22].

### 2.4. Macrocycles Containing a Bis-1-Oxaquinolizidine Moiety

#### Xestospongins/Araguspongines 

**Figure 5:**
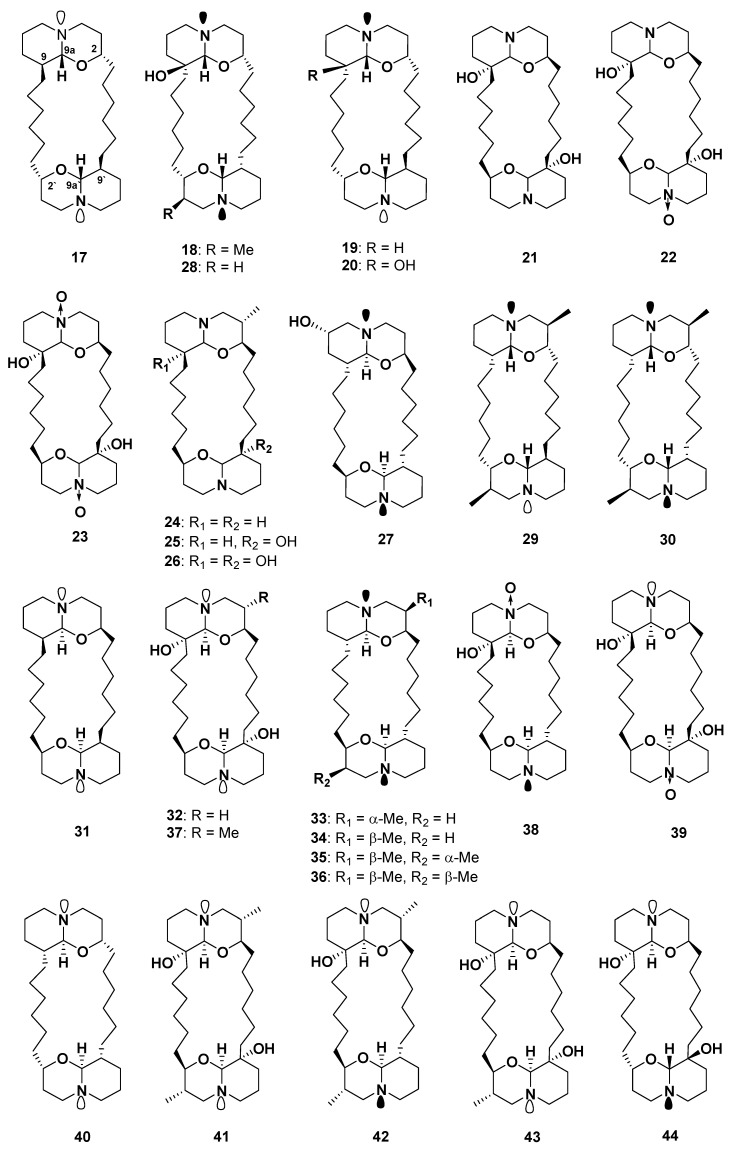
Structures of **17**–**44**.

Araguspongines (xestospongins) are a class of macrocyclic alkaloids consisting of a 20-membered ring and two 1-oxaquinolizidine moieties. Xestospongins A (araguspongine D) (**17**), B (**18**), C (araguspongine E) (**19**), and D (araguspongine A) (**20**) were isolated from the Australian sponge *Xestospongia exigua* and from *Xestospongia* sp. [17,23], whereas xestospongins E–J (**21–26**) (Figure 5) were isolated from the sponge *Oceanapia* sp. [24]. Compounds **17**–**20** were found to have an in vivo vasodilator activity [17]. In addition to this activity, **19** and **20** exhibited moderate antimicrobial activity against *Aspergillus fumigatus*, *Aspergillus niger*, *Rhodotorula*, *Candida albicans*, and *Cryptococcus neoformans* and moderate to strong antibacterial activity toward *Staphyloccus aureus* and *Escherichia coli* [24].

(+)-7*S*-Hydroxyxestospongin A (**27**) [25], demethylxestospongin B (**28**) [26], and C (**29**) were isolated from *Xestospongia* sp. [27]. Compound **28** was also isolated from *Neopetrosia exigua,* along with a quinolizidine derivative, 9′-*epi*-3β,3′β–dimethylxestospongin C (**30**) [28]. Compounds **28**–**30** showed cytotoxic activity with ED_50_ values of 0.8, 2.0, and 0.2 μg/mL against L1210 (mouse lymphocytic leukemia) and ED_50_ values of 2.5, 2.5, and 2.0 μg/mL against KB (human epidermoid carcinoma) cells, respectively [26]. 

Araguspongines B (**31**), C (**32**), F–H (**33–35**), and J (**36**) (Figure 5) were isolated from the Okinawan sponge *Xestospongia* sp. [29]. A bis-1-quinolizidine derivative, 3α-methylaraguspongine (**37**), along with **17**, **19**, **20,** and **32**, were isolated from *Xestospongia exigua* [30].

On the basis of molecular modeling and NMR spectroscopy, Hoye et al. re-examined the chemical structures of several members of araguspongine/xestospongin families of alkaloids [31]. They studied the *cis*- vs. *trans*-decalin-like conformers and the relative configuration of various substituted 1-oxaquinolizidine-containing macrocycles. They found that (i) for the unsubstituted parent compound 1-oxaquinolizidine, the *trans*-decalin-like isomer is the dominant contributor based on ^1^HNMR studies (up-field chemical shift value for the N-CH-O proton (δ 3.41), consistent with two sets of anti-periplanar non-bonding electrons to C9-Ha9, along with coupling constant values (*J*), fit the dihedral angle of trans-like isomer), and (ii) *trans*-dialkylated ring substitutions are largely common in the *trans*-decalin-like conformation, while *trans*-dialkylated ring substitutions are largely common in the *trans*-decalin-like conformation, and dialkylated ring substitutions are largely common in the *cis*-decalin-like conformation [31]. The thermodynamic stability of these conformations was due to the *trans*-dialkylated orientation and the presence of a *cis*-decalin-like structure, which provide more stability by their anomeric effect [32]. 

In 2002, two new *N*-oxide araguspongines, araguspongines K (**38**) and L (**39**), along with **17**, were isolated from the Red Sea sponge *Xestospongia exigua* [33]. Both **38** and **39** exhibited cytotoxicity against HL-60 cells with an IC_50_ value of 5.5 μM, whereas **17** showed an IC_50_ value of 5.9 μM [33]. Later on, Liu et al. isolated araguspongine M (**40**), along with **17** and **31**, from the same sponge [34]. 

Three compounds, identified as LT-9 (**41**), LT-10 (**42**), and LT-6 (**43**) (Figure 5), were isolated from the Thai water sponge *Xestospongia* sp.; however, their structures were clarified and renamed as araguspongines N−P (**41–43**) [20,35]. Araguspongines A, B, C, F, G, H, and J (**20**, **31**, **32**, **33**, **34**, **35**, and **36**) and M–P (**40**–**43**) possess bis-1-oxaquinolizidine moiety, whereas **38** and **39** have a bis-1-oxaquinolizidine *N*-oxide moiety [17,33]. The biological activities of araguspongines include antifouling, cytotoxic, antitubercular, antimalarial, somatostatin, and vasoactive intestinal peptide inhibitory effects [33,36].

Dung et al. reported the isolation of *meso*-araguspongine C (**44**) from the sponge *Xestospongia muta*. Compounds **32** and **44** showed significant cytotoxic activity against LU-1, HepG-2, HL-60, MCF-7, and SK-Mel-2 human cancer cells, with IC_50_ values ranging from 0.43 to 1.02 μM; however, **44** is more potent than **32** [20]. Compounds **20**, **32**, **38**, and **39** exhibited cytotoxicity against breast cancer BT-474 cells, with IC_50_ values of 9.3, 15.2, 29.5, and 35.6 μM, respectively [37].

Araguspongines show significant antifouling activity with low toxicity against both micro- and macrofouling organisms [33,36]. Their potent antibacterial activity has been shown against seven strains of fouling bacteria i.e., *Pseudomonas aeruginosa*, *Pseudomonas putida*, *Pseudomonas chlororaphis*, *Pseudoalteromonas haloplanktis, Bacillus cereus*, *Bacillus pumilus,* and *Bacillus megaterium* by a fraction of bis-1-oxaquinolizidine alkaloids [36].

Araguspongines that possess a macrocyclic ring with two *cis-* or *trans*-dialkylated orientations at C-2 and C-9 on both l-oxaquinolizidine rings, as well as two *trans-* or *cis*-decalin-like rings, showed potent biological activities. For example **31**, **32**, **33**, **40**, and **44** exhibited growth-inhibitory activity against HL-60, with IC_50_ values ranging from 0.62 to 5.90 μg/mL. On the contrary, compounds that have both *cis-* and *trans*-dialkylated orientation and one *cis*-decalin-like ring, or those that possess bis-1-oxaquinolizidine *N*-oxide, showed weak or no activity. This was demonstrated by the fact that **19**, **20**, and **39** exhibited weak or no biological activity against HL-60 cells, with IC_50_ values ranging from 16.79 to 22.95 μg/mL [20]. Compound **27** was inactive against foulant organisms [25]. Therefore, the stability of the aforementioned araguspongines’ conformation seems to influence their biological activity.

Compounds **19** and **20**, containing one *trans*- and one *cis*-decalin-like ring, exhibited weaker activity against HL-60 when compared to other araguspongines [26]. Compound **20** showed moderate activity relative to **18** and **28** against KB and L1210 cells. This effect might be due to the presence of the OH group at C-2 in **20** [26].

Compound **18** displaced [^3^H]IP_3_ from the membranes of cerebellar and skeletal myotube homogenates, with EC_50_ values of 44.6 ± 1.1 µM and 27.4 ± 1.1 µM, respectively [38]. This compound inhibited bradykinin-induced Ca^2+^ signals of the neuroblastoma cells (NG108-15) and selectively blocks the slow intracellular Ca^2+^ signal induced by membrane depolarization with high external K^+^ (47 mM) in rat skeletal myotubes [38]. Compound **18** decreases IP3-induced Ca^2+^ oscillations, with an EC_50_ value of 18.9 ± 1.35 µM [38]. Conclusively, **18** showed cell-permeant activity and was a competitive inhibitor of IP3 receptors in cultured rat myotubes, and it separated myonuclei and NG108-15 cells [38].

The organic extract *Haliclona exigua* exhibited adulticidal and embryostatic actions against human lymphatic filarial parasite *B. malayi* in an experimental rodent model, and this activity could be due to the presence of araguspongin C [4]. Compound **32** showed potent activity against the *Mycobacterium tuberculosis* strain H37Rv, with a minimum inhibitory concentration (MIC) value of 3.94 µM (positive control: rifampin, IC_50_ = 0.61 µM) [33].

Compound **32** displayed an in vitro anti-proliferative effect against multiple breast cancer cell lines in a dose-dependent manner. It causes the induction of autophagic cell death in HER2-overexpressing BT-474 breast cancer cells, which was characterized by vacuole formation and upregulation of autophagy markers. It displayed autophagy associated with the inhibition of c-Met and HER2 receptor tyrosine kinase activation. Compound **32** also suppressed the depression of the PI3K/Akt/mTOR signaling cascade in the breast cancer cells that undertake autophagy. The induction of autophagic death in BT-474 cells was associated with reduced levels of the inositol 1,4,5-trisphosphate receptor upon management with an effective concentration of **32** [37]. 

### 2.5. Macrocycles Containing a 3-Alkylpiperidine Moiety 

#### 2.5.1. Pentacyclic Derivatives

##### Saraines/Sarains

**Figure 6:**
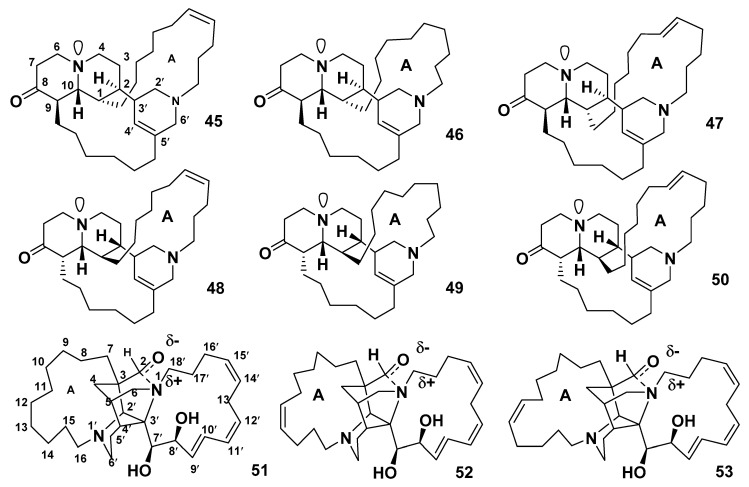
Structures of **45**–**53**.

An investigation of the marine sponge *Reniera sarai* led to the identification of saraines 1–3 (**45–47**) [39], which belong to the 3-alkylpiperidine subclass (Figure 6). The complexity of their structures delayed a complete elucidation until the mid-1980s. 

The main scaffold of saraines consists of a tetrahydropyridine moiety attached to a *trans*-2-oxoquinolizidine ring system. They possess a pentacyclic skeleton that includes a trisubstituted alkene and a carbonyl group. The two cycles are supplied by linking the two heterocyclic systems with linear alkyl chains [39]. The three stereoisomers of saraines 1–3 have been reported and identified as isosaraines 1–3 (**48–50**) [40,41,42], which were also isolated from *R. sarai* as minor components. Saraines A–C (**51–53**) were isolated from the Mediterranean sponge *R. sarai* and possess an entirely different structure from those of the previously reported saraines 1–3 (**45**–**47**) and isosaraines 1–3 (**4**8–**50**). The entire skeleton of **51–53** is composed of two piperidine rings condensed to form a central nucleus, which linked to a pair of alkyl chains [43,44]. Compounds **45**–**47** and **51**–**53** (Figure 6) exhibited antibacterial activity against *S. aureus* with MIC values between 6.25 and 50 μg/mL; a lethality against *Aspergillus salina,* with LD_50_ values between 2.5 and 46.7 μg/mL; an inhibitory effect against potato disc infected with *Aspergillus tumefaciens,* with inhibition percentages between 16% and 55%; and inhibition of the development of fertilized sea urchin eggs, with IC_50_ values between 1.56 and 6.25 μg/mL. However, **45** showed neither antimicrobial activity nor the inhibition of development of fertilized sea urchin eggs at a concentration as high as 50 μg/mL [45]. Overall, saraines show an increase in biological activity with an increase in the size of the macrocyclic ring (A) within the two groups from **45** to **47** and from **51** to **53** (Figure 6). 

##### Madangamines

**Figure 7:**
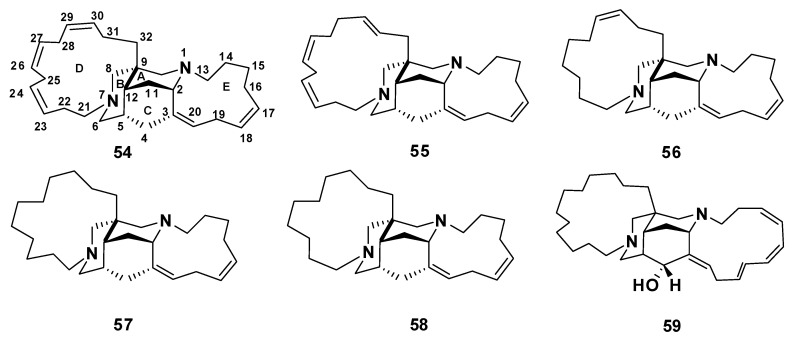
Structures of **54**–**59**.

Madangamines A (**54**) [46] and B–E (**55–58**) [47] were isolated from the marine sponge *X. ingens*, whereas madangamine F (**59**) was isolated from the sponge *Pachychalina alcaloidifera* [48]. Because of their diazatricyclic skeleton and two peripheral macrocyclic rings, madangamines have an unusual chemical structure. The macrocyclic ring D in madangamines varies in size, ranging from 13 to 15 carbon atoms. The ring E in **54**–**58** is an 11-membered ring with two double bonds, whereas **59** possesses a 13-membered ring with four double bonds [49] (Figure 7).

Compound **54** displayed significant in vitro cytotoxicity toward murine leukemia P388 (ED_50_ value of 0.93 μg/mL), lung A549 (ED_50_ value of 14 μg/mL), MCF-7 (ED_50_ value of 5.7 μg/mL), and brain U373 (ED_50_ value of 5.1 μg/mL) cancer cell lines, respectively [46]. Compound **59** showed weak cytotoxicity, with EC_50_ values of 16.7, 19.8, >25, and 16.2 µg/mL against HL-60, SF 295 (human CNS), HCT-8 (colon), and MDA-MB435 (melanoma) cancer cell lines, respectively [48].

##### Haliclonadiamines

**Figure 8:**
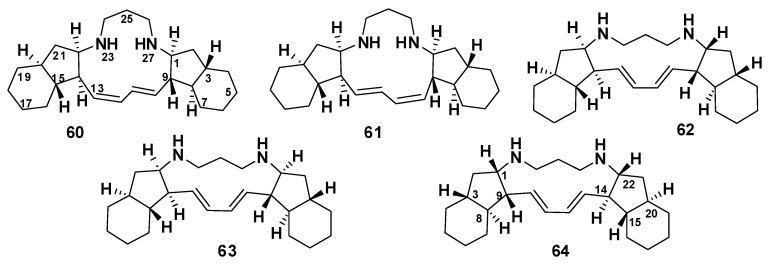
Structures of **60**–**64**.

The bis-indane macrocycles (10*E*,12*Z*)-haliclonadiamine (**60**) and (10*Z*,12*E*)-haliclonadiamine (**61**) were isolated from *Halichondria panicea* [50], whereas papuamine (**62**) [51] and haliclonadiamine (**63**) [52] were isolated from *Haliclona* sp. Compounds **60**–**63** showed a potent effect against *Mycobacterium smegmatis* with inhibitory zones of 7–16 mm at a concentration of 10 μg/disc [53]. Compound **63** exhibited a potent effect with an inhibition zone of 16 mm at 10 μg/disc. SAR analysis suggests that the antitubercular activity of these compounds favors the 13-membered ring E and the 10*E*,12*E* configuration [53] (Figure 8). Recently, Liu et al. have revised the structure of **63** using X-ray crystallography, establishing the absolute configurations of the stereogenic carbons as 1*S*,3*R*,8*S*,9*R*,15*S*,20*R*,22*R* (**64**), which are opposite to those previously reported for **63** [54].

##### Ingenamines and Ingamines

**Figure 9:**
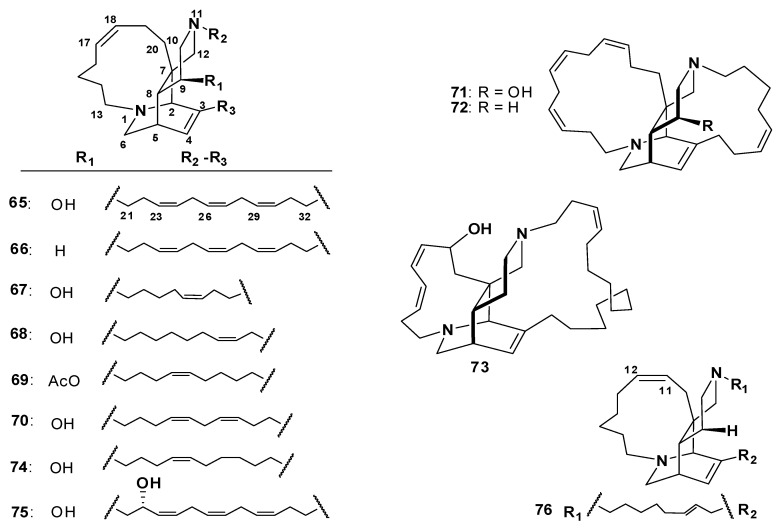
Structures of **65**–**76**.

Ingamines A (**65**) and B (**66**) [55], ingenamine A (**67**) [56], and ingenamines B–F (**68–72**) [57] were all isolated from *X. ingens*, whereas ingenamine G (**73**) was isolated from the sponge *Pachychalina* sp. [8]. Meanwhile, dihydroingenamine D (**74**) and 22(*S*)-hydroxyingamine A (**75**) were isolated from the sponge *Petrosid Ng5 Sp5* [58] (Figure 9). Compounds **63**, **74**, and **75** exhibited antiplasmodial activity against chloroquine-resistant (W2) and chloroquine-sensitive (D6) strains of *Plasmodium falciparum,* with IC_50_ values of 57 and 72 ng/mL for **63**, 78 and 90 ng/mL for **74**, and 140 and 200 ng/mL for **75**, respectively [58]. Compound **73** exhibited cytotoxic activity, with IC_50_ values of 11.3, 9.8, and 8.6 µg/mL against MCF-7, B16 (leukemia), and HCT-8 cancer cells, respectively [8]. Moreover, this compound showed antimicrobial activity with MIC values at 8 µg/mL against *M. tuberculosis* H37Rv, 105 µg/mL against *S. aureus* (ATCC 25923), 75 µg/mL against *E. coli* (ATCC 25922), and with MIC values ranging from 10 to 50 µg/mL against two of four strains of oxacillin-resistant *S. aureus* [8]. Xestocyclamine (**76**) is a *pseudo*-enantiomeric to **67**, and they differ only in the location of the carbon–carbon double bond in the 11-membered ring. Compound **76** exhibited moderate inhibitory activity against protein kinase C, with an IC_50_ value of 4 µg/mL. Interestingly, **76** showed selectivity against IL-1 (interleukin), as it showed no activity against other cancer-relevant targets [59]. 

#### 2.5.2. Tetracyclic Derivatives

##### Halicyclamines

**Figure 10:**
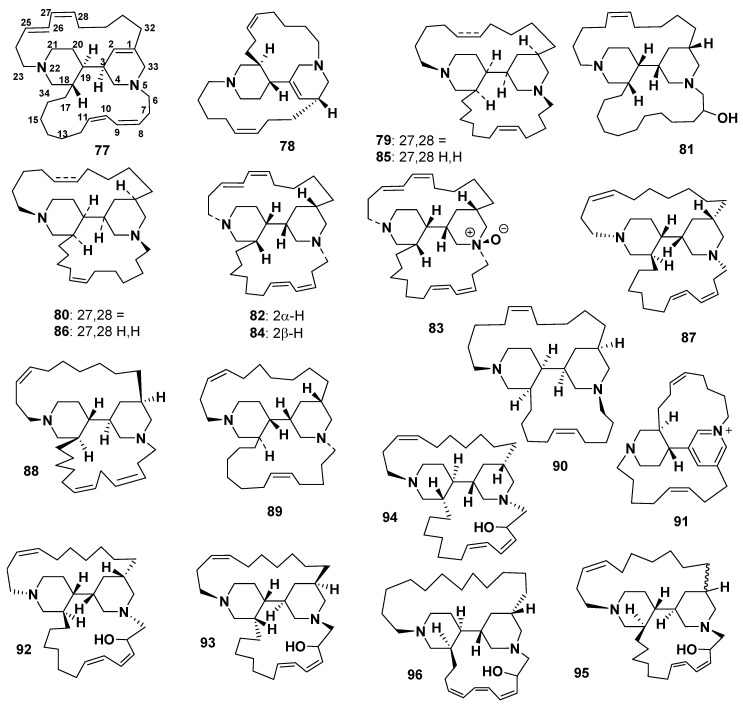
Structures of **77**–**96**.

Halicyclamines A (**77**) and (-) halicyclamine B (**78**) were isolated from *Haliclona* sp. [60] and *Xestospongia* sp. [61], respectively (Figure 10). Haliclonacyclamines A (**79**) and B (**80**) [62] were isolated from *Haliclona* sp. 22-Hydroxyhaliclonacyclamine B (**81**) [63], 2-*epi*-tetradehydro haliclonacyclamine (**82**), tetradehydrohaliclonacyclamine A mono-*N*-oxide (**83**), and tetradehydrohaliclonacyclamine A (**84**) were isolated from *Halichondria* sp. [64]. The anti-dormant mycobacterial activity of **77** was reported by Kobayashi et al., with the correlation of Ded A Protein to the mechanism of action of **77** under dormancy-inducing hypoxic and standard aerobic growth conditions [65]. Compound **78** showed weak and selective antimicrobial activity and also exhibited growth inhibitions of 50% and 20% at 200 μg/disk against *Bacillus subtilis* and *E. coli,* respectively, but showed no activity toward *C. albicans* [61]. Compound **79**, isolated from the *Haliclona* sponge of the Solomon Islands, exhibited a great antiplasmodial effect in vivo and in vitro against *Plasmodium vinckei petteri*-infected mice and the chloroquine-resistant *P. falciparum* strain FCB1. It also shows IC_50_ values of 0.052 and 0.33 μg/mL against the *P. falciparum* strain FCB1 and chloroquine-sensitive 3D7, respectively [66]. In vitro, **79** displayed cytotoxicity against MCF-7 cells (2.6 μg/mL) [66].

Haliclonacyclamines C (**85**) and D (**86**) were isolated from a specimen of *Haliclona* sp. collected from Heron Island on the Great Barrier Reef [67].

Haliclonacyclamine E (**87**) was isolated from the Haplosclerida sponge *Arenosclera brasiliensis,* which is endemic to the Southeastern coast of Brazil [68]. Compound **87** displayed cytotoxicity against HL60, B16, L929 (brosarcoma), and U-138 (colon) cancer cell lines, with IC_50_ values of 4.23, 1.82, 3.89, and 6.06 μg/mL, respectively [69]. Haliclonacyclamine F (**88**) was isolated from the sponge *P. alcaloidifera*. Compound **88** exhibited cytotoxicity against HL-60, SF 295, HCT-8, and MDA-MB435 cancer cell lines with IC_50_ values of 2.2, 4.5, 8.6, and 1.0 µg/mL, respectively [48]. Halichondramine (**89**) was isolated from the Red Sea sponge *Halichondria* sp. [70].

A bis-piperidine alkaloid, neopetrosiamine A (90), isolated from *Neopetrosia proxima*, showed potent inhibitory activity against MCF-7, CCRF-CEM (leukemia), and MALME-3M melanoma cancer cells, with IC_50_ values of 3.5, 2.0, and 1.5 μΜ, respectively. Compound **90** also exhibited in vitro cytotoxicity, with an MIC value of 7.5 μg/mL, toward a pathogenic strain of *M. tuberculosis* (H_37_Rv) in a microplate Alamar Blue assay (MABA). Additionally, **90** showed antiplasmodial activity against *P. falciparum,* with an IC_50_ value of 2.3 μM [71]. Although **78** and **90** have very similar structural features, with one of the alkyl chains of **90** being shorter than that of **78** and exhibiting stronger activity against *P. falciparum* than **78**, **78** showed higher activity than **90** against MCF7 breast cancer cells [71].

Tetradehydrohalicyclamine B (**91**) and **78** were isolated from the sponge *Acanthostrongylophora ingens.* Both compounds showed inhibition against the constitutive proteasome and immunoproteasome. Compound **78** revealed 4- to 10-fold higher inhibitory activity than **91** [72].

##### Arenosclerins

Arenosclerins A–C (**92–94**) were isolated from the Brazilian endemic Haplosclerida sponge, *A. brasiliensis* [68], whereas arenosclerins D (**95**) and E (**96**) (Figure 10) were isolated from the sponge *P. alcaloidifera* [48]. Although these compounds were inactive against *C. albicans*, **92** and **94** showed antibacterial activity against a larger number of bacteria strains than **93**; however, potent antibacterial activity was exhibited by both **93** and **94**. Moreover, these compounds showed potent toxicity toward HL-60, B16, L929, and U-138 cancer cell lines [69]. The IC_50_ values of **92** were 1.77, 2.34, 4.31, and 3.83 μg/mL; of **93** were 1.76, 2.24, 4.07, and 3.62 μg/mL; and of **94** were 1.71, 2.17, 3.65, and 3.60 μg/mL against B16, L929, HL-60, and U-138 cancer cell lines, respectively [69].

Compounds **95** and **96** were tested for their cytotoxicity against HL-60, SF 295, HCT-8, and MDA-MB-435 cancer cell lines, and their IC_50_ values were 2.1, 5.9, 6.2, and 1.2 µg/mL and 6.9, 8.7, >25, and 3.1 µg/mL, respectively [48].

### 2.6. Manzamines

#### 2.6.1. Pentacyclic Manzamines

Pentacyclic manzamines are a group of macrocyclic alkaloids containing a β-carboline moiety attached to pentacyclic rings with a double bond between C-10 and C-11 in the eight-membered ring [73,74]. 

Manzamine A hydrochloride salt (**97**), the first reported member of manzamines, was isolated from *Haliclona* sp. [75]. This compound was also isolated from *Pellina* sp. and was named keramamine A [76]. Compound **97** showed a broad spectrum of biological effects, i.e., potent antipathogenic activity against *Leishmania donovani*, antimycobacterial activity [77], cytotoxicity against pancreatic cancer (by inhibiting autophagy) [78], P388 [75], human colorectal carcinoma [79], and anti-Alzheimer activity [80]. It also exhibited an inhibitory effect against herpes simplex virus (HSV-1) [81] and HSV-2 [82], human immunodeficiency virus (HIV) [77], as well as the rodent malaria parasite *Plasmodium berghei* in vivo [10].

**Figure 11:**
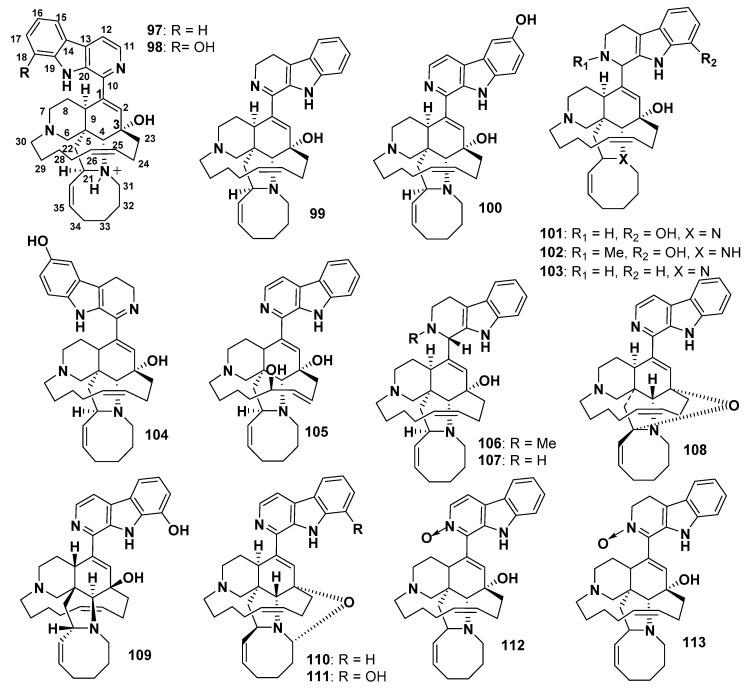
Structures of **97**–**113**.

8-Hydroxymanzamine A (**98**, also known as manzamine G or manzamine K) was isolated from *Pachypellina* sp. and the stereochemistry of **98** was the same as **97** (Figure 11), as both of them were dextrorotatory. Compounds **97** and **98** exhibited moderate antitumor activity against KB and LoVo (colon) cancer cell lines and anti-HSV-II (herpes simplex) activity [82]. Compounds **97** and **98** displayed in vitro and in vivo antimalarial effects against *P. berghei.* The percentage of the asexual erythrocytic stages suppression, which registered after a single intraperitoneal injection of **97** and **98** administered to infected mice, was 90%. These compounds increased the time of living of the infected mice to more than 240 h, using just one dose of **97** (50 mM/kg) and **98** (100 mM/kg) [83].

3,4-Dihydromanzamine A (**99**) and 6-hydroxymanzamine A (manzamine Y) (**100**), isolated from a marine sponge *Amphimpdon* sp., showed antibacterial activity against a Gram-positive bacterium, *Sarcina lutea* (MIC values of 4 and 1.25 µg/mL, respectively). These compounds also exhibited in vitro cytotoxicity against L1210 (IC_50_ values of 0.48 and 1.5 µg/mL, respectively) and KB cells (IC_50_ values of 0.61 and 2.5 µg/mL, respectively) [84].

1,2,3,4-Tetrahydro-8-hydroxymanzamine A (8-hydroxymanzamine D) (**101**), and 1,2,3,4-tetrahydro-2-*N*-methyl-8-hydroxymanzamine A (8-hydroxy-2-*N*-methylmanzamine D) (**102**) (Figure 11) were isolated from the marine sponges of the genera *Petrosia* and *Cribochalina* [85]. Compound **102** is cytotoxic toward P388 cell line, with an ED_50_ value of 0.8 µg/mL [85]. Manzamine D (1,2,3,4-tetrahydromanzamine A) (**103**) was isolated from *Ircinia* sp. [86], whereas 3,4-dihydro-6-hydroxymanzamine A (**104**) and manzamine M (**105**) were isolated from *Amphimedon* sp. [87]. Compound **105** was the first reported manzamine congener with a hydroxyl group on the C13-C20 chain. Compounds **104** and **105** showed cytotoxicity against L1210 cells (IC_50_ values of 0.3 and 1.4 µg/mL, respectively). Moreover, **104** and **105** exhibited antibacterial activity against *Sarcina lutea* (MIC values of 6.3 and 2.3 µg/mL, respectively) and *Corynebacterium xerosis* (MIC values of 3.1 and 5.7 µg/mL, respectively) [87]. Bioassay-directed fractionation of the CH_2_Cl_2_ crude extract of the Palaun sponge, employing an assay for the inhibitors of methionine aminopeptidase-2 (Met AP-2), led to the identification of *N*-methyl-*epi*-manzamine D (**106**) and *epi*-manzamine D (**107**) [88]. Neither of these compounds exhibited selectivity in the yeast assay for inhibitors of Met AP-2; however, both compounds showed cytotoxicity against HeLa and B16F10 melanoma cells. Compound **106** showed strong activity against the B16F10 cell line [88]. 12,34-Oxamanzamine A (**108**) was isolated from an Indo-Pacific sponge identified as 011ND 51 [89]. This compound possesses an unusual ring system due to the presence of an ether bridge formed between C-12 and C-34 of the typical manzamine structure. Compound **108** displayed less activity against malaria and the AIDS OI pathogen, *M. tuberculosis*, compared to the other co-isolated manzamines, which might be attributed to the presence of the C12–C34 ether bridge in **108** [89] (Figure 11). *ent*-8-Hydroxymanzamine A (**109**) was isolated from an undescribed genus of an Indo-Pacific sponge. It exhibited improved activity against P-388, with an IC_50_ value of 0.25 µg/mL [90]. Compound **109** displayed in vitro growth inhibitory effect against *Trypanosoma gondii* and host cell with 71% and 38% inhibition, respectively, at a concentration of 1 µM [90]. 12,28-Oxamanzamine A (**110**) and 12,28-oxa-8-hydroxymanzamine A (**111**) were isolated from two collections of an Indo-Pacific sponge. These compounds contain a novel manzamine-type ring system, generated through a new ether bridge formed between C-12 and C-28 or between C-12 and C-34 of the typical manzamine structure. These compounds exhibited potent anti-inflammatory, antifungal, and anti-HIV-1 activities [91].

Manzamine A *N*-oxide (**112**) and 3,4-dihydromanzamine A *N*-oxide (**113**) were isolated from the Indonesian marine sponge *Xestospongia ashmorica* [92]. Compound **112** showed potent cytotoxicity against L5178Y mouse lymphoma cells with an ED_50_ of 1.6 µg/mL [92]. 

**Figure 12:**
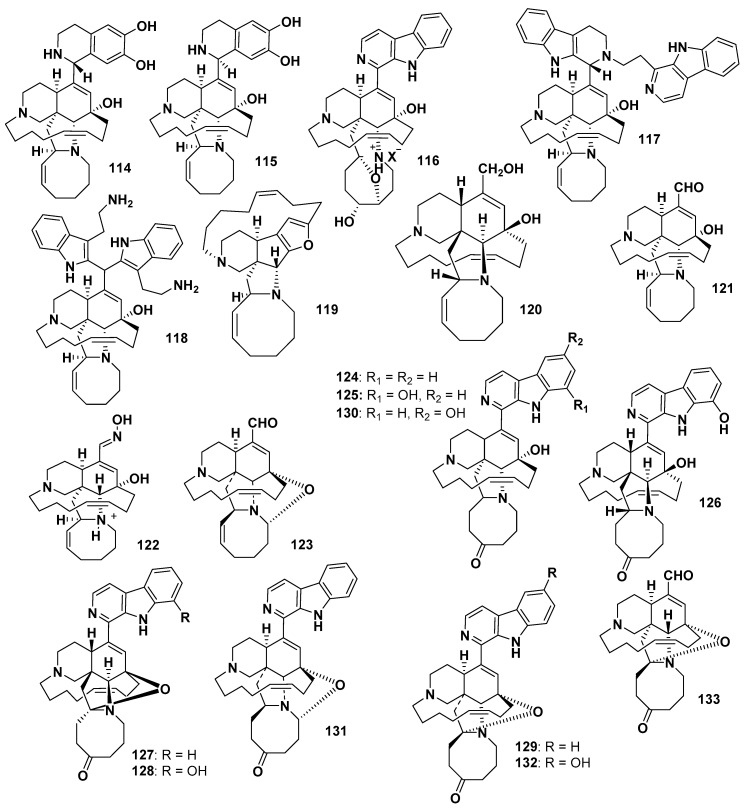
Structures of **114**–**133**.

Acanthomanzamines A (**114**) and B (**115**), isolated from *A. ingens,* contain a tetrahydroisoquinoline ring system instead of β-carboline. Compounds **114** and **115** showed potent cytotoxicity against HeLa cells, with IC_50_ values of 4.2 and 5.7 μM, respectively. Interestingly, **114** and **115** (Figure 12) exhibited stronger cytotoxicity against HeLa cancer cell line, but less potent proteasome inhibitory activity than their co-isolated β-carboline-containing manzamines, acanthomanzamines D and E [93]. Several other examples of β-carboline-based manzamines were also reported from different sponge species. Examples of these are pre-*neo*-kauluamine (**116**) from *A. ingens* [94], zamamidine C (**117**) [95], zamamidine D (**118**) [96], nakadomarin A (**119**) from *Amphimedon* sp. [97], ircinol A (**120**) from *Amphimedon* sp. [98], ircinal A (**121**) from *Ircinia* sp. [86], ircinal E (**122**) from *A. ingens* [99], and 12,28-oxaircinal A (**123**) from *Acanthostrongylophora* sp. [100]. The reported biological activities of the aforementioned compounds were quite interesting, Compound **116** showed proteasome inhibitory activity [94], whereas **117** displayed potent antitrypanosomal effect against *Trypanosoma brucei brucei* and antimalarial activity against *P. falciparum* [95]. Compound **118** exhibited antimicrobial activity against several strains of fungi and bacteria [96], whereas **119** exhibited antimicrobial effects against *C. xerosis* and *Trichophyton mentagrophytes,* with MIC values of 11 and 23 µg/mL, respectively [97]. Compound **120** inhibited endothelin-converting enzyme, with an IC_50_ of 55 µg/mL [98]. Compound **121** displayed cytotoxicity against L1210 and KB cancer cells with IC_50_ values of 1.4 and 4.8 µg/mL, respectively [86]. Compound **122** showed weak cytotoxicity and L5178Y (murine lymphoma) cells with an IC_50_ value of 21.7 µg/mL, respectively [99]. Pentacyclic manzamines having a ketonic group in their eight-membered ring instead of a double bond were also reported. Examples of this class of compounds are manzamines E (**124**) [76], F (keramamine B) (**125**) from *Xestospongia* sp. [101], *ent*-manzanine F (**126**) from *Petrosia* sp. [90], *ent*-12,34-oxamanzamines E (**127**) and F (**128**) from the sponge *011ND 35* [89], 12,34-oxamanzamine E (**129**) and 6-hydroxymanzamine E (**130**) from *Acanthostrongylophora* sp. [77], 12,28-oxamanzamine E (**131**) and 12,34-oxa-6-hydroxymanzamine E (**132**) from *Acanthostrongylophora* sp. [100], and the related manzamine alkaloid 31-keto-12,34-oxa-32,33-dihydroircinal A (**133**) from the marine sponge of the genus 011ND 35 [91] (Figure 12). Compounds **124** and **125** displayed cytotoxicity toward L5178Y cells, with ED_50_ values of 6.6 and 2.3 µg/mL), respectively [92], whereas they showed similar significant cytotoxicity against P388 cells with an IC_50_ value of 5.0 µg/mL [101]. Compound **126** inhibited *M. tuberculosis* (H37Rv) with an IC_50_ < 12.5 µg/mL [90]. Compound **127** showed weak inhibitory activity against *M. tuberculosis* with an IC_50_ value of 128 µg/mL, whereas **128** showed significant activity with IC_50_ 12.5 µg/mL [89].

#### 2.6.2. Tetracyclic Manzamines

**Figure 13:**
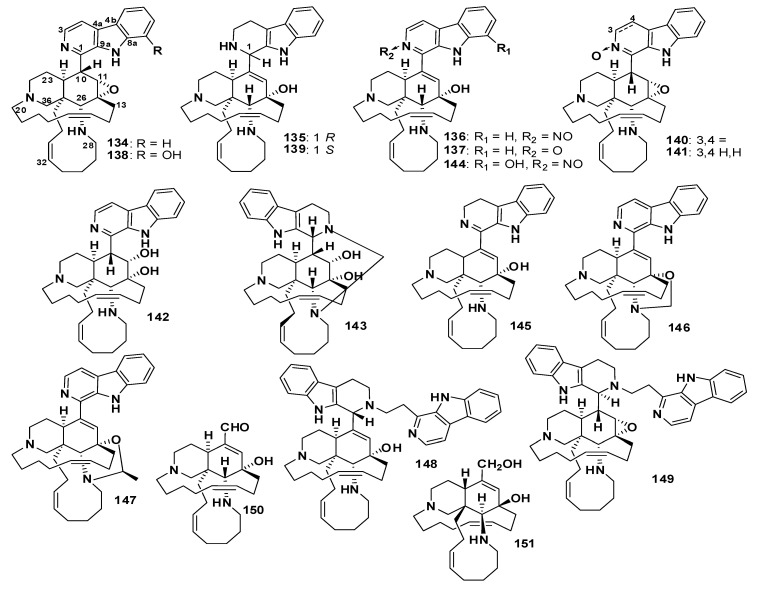
Structures of **134**–**151**.

Several manzamines containing a β-carboline ring system linked to a tetracyclic scaffold have been reported. For example, manzamine B (**134**) was reported from *Haliclona* sp. [102], manzamines H (**135**) and J (**136**) were isolated from *Ircinia* sp. [86], manzamine J *N*-oxide (**137**) was reported from *X. ashmorica* [92], 8-hydroxymanzamine B (**138**) was reported from *Acanthastrongylophora* sp. [100], manzamine L (**139**) was published from *Amphimedon* sp. [103], manzamine B *N*-oxide (**140**), 3,4-dihydromanzamine B *N*-oxide (**141**) and 11-hydroxymanzamine J (**142**) were reported from *Acanthastrongylophora* sp. [104], ma’eganedin A (**143**) was isolated from *Amphimedon* sp. [105], 8-hydroxymanzamine J (**144**) was reported from *Acanthastrongylophora* sp. [77], 3,4-dihydromanzamine J (**145**) was isolated from *Amphimedon* sp. [87], acanthomanzamine D (**146**) and acanthomanzamine E (**147**) were reported from *A. ingens* [93], zamamidines A (**148**) and B (**149**) were reported from *Amphimedon* sp. [106], ircinal B (**150**) was published from *Ircinia* sp. [86], and ircinol B (**151**) was reported from *Amphimedon* sp. [98] (Figure 13).

Compounds **135**, **136**, **139**, **143**, **145**, **150,** and **151** showed cytotoxic activity against L1216 cancer cell line with IC_50_ values of 1.3, 2.6, 3.7, 4.4, 5.0, 1.9, and 7.7 µg/mL, respectively. Furthermore, **135**, **136**, **139**, **150**, and **151** displayed cytotoxicity against KB cancer cells with IC_50_ values of 4.6, >10, 11.8, 3.5, and 9.4 µg/mL, respectively, whereas **137** showed cytotoxicity against L1578Y with IC_50_ values of 1.6 µg/mL, and **148** and **149** showed cytotoxic activity against P388 cells with IC_50_ values of 13.8 and 14.8 µg/mL, respectively. Compounds **146** and **147** displayed a strong proteasome inhibitory effect, with IC_50_ values of 0.63 and 1.5 µg/mL, respectively [93]. Compounds **139** and **140** showed weak activity against several Gram-positive and Gram-negative bacteria [104]. Compound **143** showed potent activity against *Sarcina lutea* and *B. subtilis,* with the same MIC value of 2.8 µg/mL [105]. The reported antimicrobial activity of several manzamines highlights the influence of an eight-membered ring on the activity [77]. Moreover, the antitubercular activity is also affected by the ring size; for example, compounds **97** and **136** have similar scaffold, except eight-membered ring in **97** and 11-membered in **136** [83]. Compound **97** exhibited potent anti-tubercular activity against *M. tuberculosis* (H37Rv) than **136** [83].

#### 2.6.3. Monomacrocycle Containing Manzamines and Related Compounds

**Figure 14:**
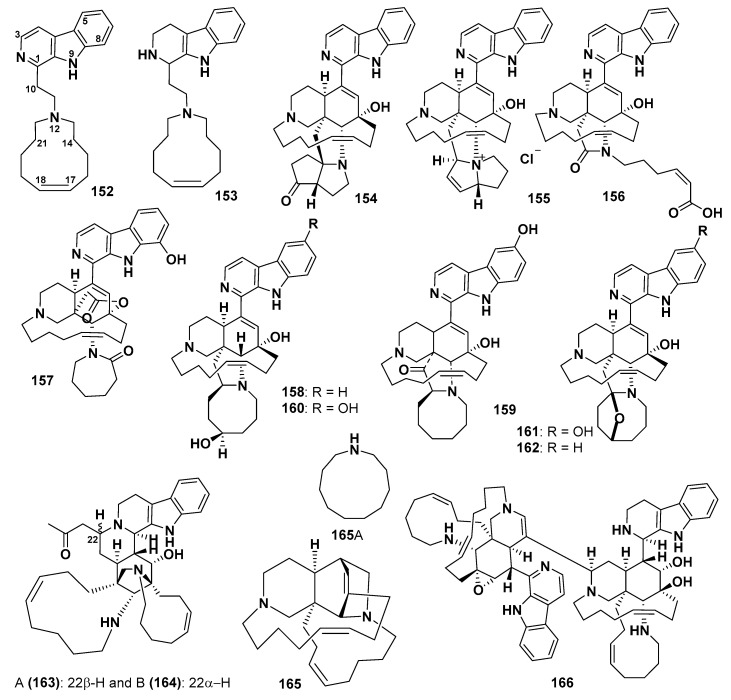
Structures of **152**–**166**.

Compounds in this group have one macrocyclic ring of different sizes, namely, 10-, 11-, 13-, 14- and 15-membered rings. Manzamine C (**152**) was initially isolated from the Okinawan sponge *Haliclona* sp. This compound possesses an 11-membered heterocyclic ring containing a nitrogen atom [102]. Compound **152** exhibited cytotoxicity against A549, HT-29, and P-388 cells with IC_50_ values of 3.5, 1.5, and 2.6 μg/mL, respectively [107]. The other manzamine alkaloids containing one macrocyclic ring are keramamine C (**153**) [108], acanthomanzamine C (**154**) [93], kepulauamine A (**155**) [104], acantholactam (**156**) [94], and acantholactone (**157**) [109] (Figure 14). Compound **153** was isolated from the Okinawan marine sponge *Amphimedon* sp. [108] and was probably a biogenetic precursor of **152**. Compound **154** was isolated from *A. ingens* [93] and was recorded as one of the first examples of a manzamine-related alkaloid containing a tetrahydroisoquinoline ring system rather than a β-carboline moiety. The hexahydrocyclopenta [*b*]-pyrrol-4(2*H*)-one ring in **154** could have originated from an eight-membered ring in manzamine A (**97**). Compound **155** was isolated from an Indonesian marine sponge, *Acanthostrongylophora* sp. This compound contains a pyrrolizine ring system, which is unique among the manzamines. It exhibited weak inhibition against K562 (human erythroleukemic) and A549 cells and is moderately active against diverse strains of pathogenic bacteria. However, this compound is inactive against sortase A (SrtA) and Na^+^/K^+^-ATPase [104]. Compound **156** was isolated from *A. ingens* and contains a γ-lactam ring with a 2Z-hexenoic acid substituent on the nitrogen atom and is proposed to be biosynthetically derived from compound **97**. It shows no proteasome inhibitory activity [94].

Acantholactone (**157**), a manzamine-related scaffold with unique δ-lactone and ε-lactam rings, was reported from *Acanthostrongylophora* sp. The absolute configurations of the stereogenic carbons of **157** were determined as 12*S*, 24*R*, 25*R*, and 26*R* by comparison of calculated and experimental electronic circular dichroism (ECD) spectra [109].

32,33-Dihydro-31-hydroxymanzamine A (**158**), 32,33-dihydro-6-hydroxymanzamine A-35-one (**159**), and 32,33-dihydro-6,31-dihydroxymanzamine A (**160**) were isolated from an unidentified Indonesian sponge [110]. Compounds **158** and **159** showed no effect against malaria and leishmanial [110]. Rao et al. reported that the decrease of antimalarial activity is attributed to the reduction of the C32-C33 double bond and oxidation of C31 [110].

Manzamine X (**161**) was reported from *Xestospongia* sp. Compound **161** exhibited cytotoxic activity against KB cells, with an IC_50_ value of 7.9 μg/mL [111].

6-Deoxymanzamine X (**162**) was isolated from *Xestospongia ashmorica* [92]. Compound **162** showed cytotoxicity against the L5178 cells with ED_50_ value of 1.8 µg/mL, and exhibited a growth-inhibitory effect against *Spodoptera littoralis* larvae with a percentage of lethality of 18.8% at a dose of 132 ppm [92].

Manadomanzamines A (**163**) and B (**164**) were reported from the Indonesian sponge, *Acanthostrongylophora* sp. [112]. These compounds exhibited tubercular effect against *Mycobacterium tuberculosis,* with MIC values of 1.9 and 1.5 µg/mL, respectively. Rifampin was used as a control and showed tubercular effect with MIC values of 0.16 µg/mL. Compounds **163** and **164** showed cytotoxic activity against HIV-1, with EC_50_ values of 7.0 and 16.5 µg/mL, respectively. Compound **163** was cytotoxic against A-549 and HCT-116 cells, with IC_50_ values of 2.5 and 5.0 µg/mL, respectively, whereas **164** was cytotoxic against HCT-116, with an IC_50_ value of 5.0 µg/mL. Compounds **163** and **164** were not cytotoxic against the normal Vero cell line at a concentration of 4.8 µg/mL. Compound **164** exhibited antifungal effect against *Cryptococcus neoformans,* with MIC value of 3.5 µg/mL, whereas **163** exhibited antifungal activity against *Candida albicans* with MIC value of 20 µg/mL [112].

Keramaphidin B (**165**), an unprecedented pentacyclic manzamine, was isolated from *Amphimedon* sp. (Figure 14). Compound **165** exhibited cytotoxic effect against P-388 and KB cells, with IC_50_ values of 0.28 and 0.3 µg/mL, respectively [113].

Kauluamine (**166**), a manzamine dimer, was isolated from the Indonesian sponge *Prianos* sp. [114]. Compound **166** exhibited a moderate immunosuppressive effect in a mixed lymphoma reaction [114].

#### 2.6.4. Structure–Activity Relationship (SAR) of Manzamine Derivatives on Antimalarial Activity

Manzamines exhibited potent antimalarial activity due to their multifunctionality scaffold. Thus, an overview of the structure–activity relationships (SARs) of manzamines as antimalarial agents can be summarized. The presence of β-carboline and pentacyclic ring systems played an important role in the antimalarial activities. The absence of these rings, for example in iricinal scaffold, led to decreasing the antimalarial activity. 9-*N* alkylation of the β-carboline ring led to decreasing antimalarial activity, whereas 9-*NH* increased the activity. Hydroxyl group substitution of the β-carboline ring, particularly position 8, exhibited no effect as antimalarial. Substitution of the nitro or methoxy groups at position 6 led to slight effects as antimalarial, while it was retained upon substitution of a methyl ester at position 3 of the β-carboline. The conformational of β-carboline played a vital role in antimalarial activity of manzamines. Modification of the planarity of β-carboline by changing pyridine into piperidine and 2-*N*-methylation led to reduction of antimalarial activity. An amide substitution on positions 8 and 6 of the β-carboline ting system reduced antimalarial activity. A 2-*N*-oxide derivative of manzamine A reserves its antimalarial potency, whereas 2-*N*-methylation of manzamine A decreased antimalarial potency against D6 and W2 strains, respectively. The hydroxyl group at C-12 was essential for antimalarial activity. The structure of manzamine F was connected to the potent antimalarial effect of 8-hydroxymanzamine-A, with a carbonyl group at C-31 and a reduced C-32 double bond, exhibiting a reduction in antimalarial activity. Modification of the C-31 C=O to a hydrazone and alkylation greatly improves the antimalarial effect. Reduction of the carbonyl group at position 31 or introduction of a double bond in conjugation with the carbonyl group (C-31) showed no antimalarial activity. A double bond at carbon-31 in an eight-membered ring was required to maintain the integrity of the ring system and thereby played an important role in contributing to antimalarial activity. Saturation of the double bond at C-31 affects the integrity of the ring and resulting in a significant reduction in antimalarial activity, while a successive reduction of the double bond at C-15 increases antimalarial activity [83].

### 2.7. Macrocycles Containing 3-Alkyl Pyridinium Salts

#### 2.7.1. Cyclostellettamines

**Figure 15:**
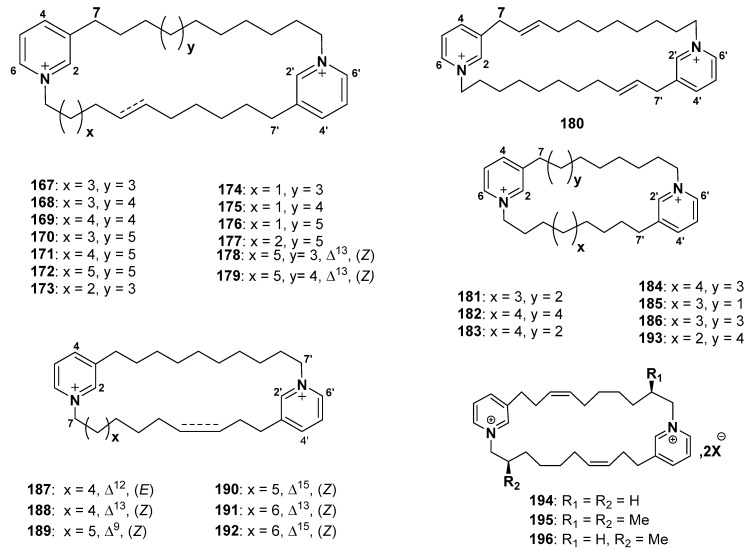
Structures of **167**–**196**.

Cyclostellettamines A–F (**167–172**) were reported from *Stelletta maxima* [115] and *Pachychalina* sp. [8]. Cyclostellettamines G–I (**173–175**), K (**176**), and L (**177**) were isolated from the marine sponge *Pachychalina* sp. [8] (Figure 15). Compounds **167**–**177** exhibited antimicrobial activity against *Candida albicans* ATCC 10231, *S. aureus* ATCC 25923, *Pseudomonas aeruginosa* strain P1, *E. coli* ATCC 25922, *P. aeruginosa* ATCC 27853 (strain Pa), oxacillin-resistant *S. aureus,* and oxacillin-resistant *S. aureus,* whereas **168**, **169**, **173**, and **177** showed potent activity against *M. tuberculosis* H37Rv (MtH37Rv) [116]. Cyclostellettamine C (**169**) was the most potent antimicrobial activity among all investigated Cyclostellettamines. With the exception of *E. coli* ATCC 25922 (Ec) and *S. aureus* ATCC 25923 (Sa), the antimicrobial activity of these cyclostellettamines is suggested to be influenced by the size of the alkyl chains [116]. Dehydrocyclostellettamines D (**178**), E (**179**), and cyclostellettamine G (**173**) were reported from the sponge of the genus *Xestospongia* [117]. These compounds showed moderate inhibitory activity against histone deacetylase from K562 cells with IC_50_ values of 17, 30, and 80 μM. Compounds **178**, **179**, and **173** exhibited cytotoxic activities against P388 cells with IC_50_ values of 1.3, 1.3, and 2.7 μM; against HeLa cells with IC_50_ values of 0.60, 1.8, and 2.8 μM; and against 3Y1 (rat fibroblastic cells) IC_50_ values of 4.3, 3.2, and 11 μM [117], respectively. Xu et al. isolated 8,8ʹ-dienecyclostellettamine (**180**) from the sponge *Amphimedon compressa*. **180** exhibited strong potent antibacterial activity [118].

Cyclostellettamines N (**181**), R (**182**), O (**183**), and Q (**184**) were reported from *Haliclona viscosa* [119]. Eight cyclostellettamine derivatives (**185–192**) were reported from *Haliclona* sp., without given specific names [120]. Compounds **181** and **184**–**192** exhibited moderate cytotoxicity against A549 cancer cell lines, whereas **184**, **186**, and **190**–**192** showed strong antibacterial activity against a number of Gram-positive and Gram-negative bacteria [120]. Lee et al. studied the effect of degree of saturation, the length of the alkyl chains, and the double-bond locations effects on the biological activities of the compounds **184**, **186**, and **190**–**192**, and they found that the biological activities were influenced by (i) the length of the alkyl chains, (ii) the distance between the charged groups, and (iii) the electron-rich locations [120].

In 2017, cyclostellettamine P (**193**) with C9 and C11 alkyl chains was detected by ion mobility–mass spectrometry [121] (Figure 15).

#### 2.7.2. Njaoaminiums

Cyclic 3-alkylpyridinium salts, njaoaminiums A (**194**), B (**195**), and C (**196**) are alkylpyridinium salts (proposed to be the precursor of njaoamine alkaloids) reported from *Reniera* sp. [122] (Figure 15). Compound **195** exhibited growth inhibitory activity against MDA-MB-231, A549, HT29 with GI_50_ values of 4.8, 4.1, and 4.2 μM [122].

### 2.8. Motuporamines

**Figure 16:**
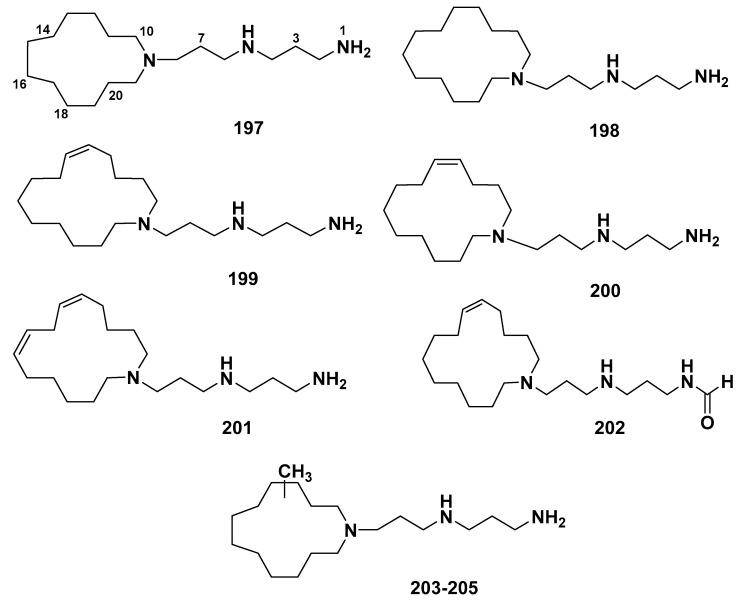
Structures of **197**–**205**.

Motuporamines A-C (**197**–**199**) (Figure 16) [123], were isolated from the marine sponge *X. exigua*. Later on, three new motuporamines D–F (**200**–**202**), a mixture of motuporamines G–I (**203**–**205**) (Figure 16) along with compounds **197**–**199,** were isolated from the same marine sponge [124]. This subclass was characterized by the presence of a saturated macrocyclic ring of the 13 to 15 carbons and two basic nitrogen atoms in the linear side chain. Compounds **197**–**199** and **203**–**205** exhibited significant anti-invasion effects, with IC_50_ values less than 15 µM, whereas no anti-invasion activity was shown by **200** and **201** [124]. The SARs explained the importance of the saturated 15-membered cyclic amine, which fused to the motuporamines diamine side chain, as the required structure for anti-invasive effects [124].

## 3. Biosynthetic Considerations

**Figure 17:**
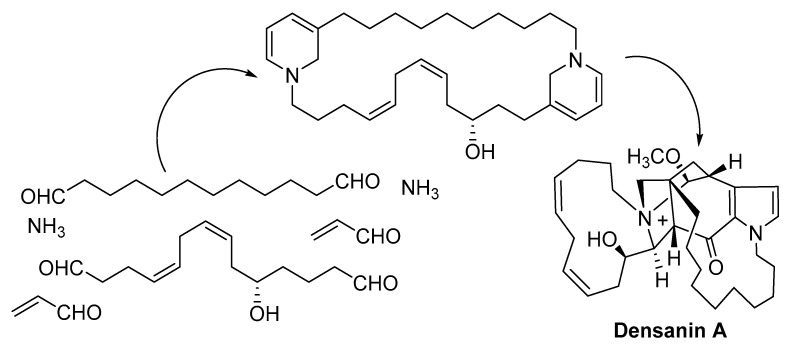
Plausible biosynthetic pathway of densanin A.

Densanin A (**1**) was a unique alkaloid and was characterized by a hexacyclic diamine skeleton with two long chains. Figure 17 shows a plausible biosynthetic pathway of densanin A from 3-alkylpyridine, as proposed by Baldwin and Whitehead [125].

**Figure 18:**
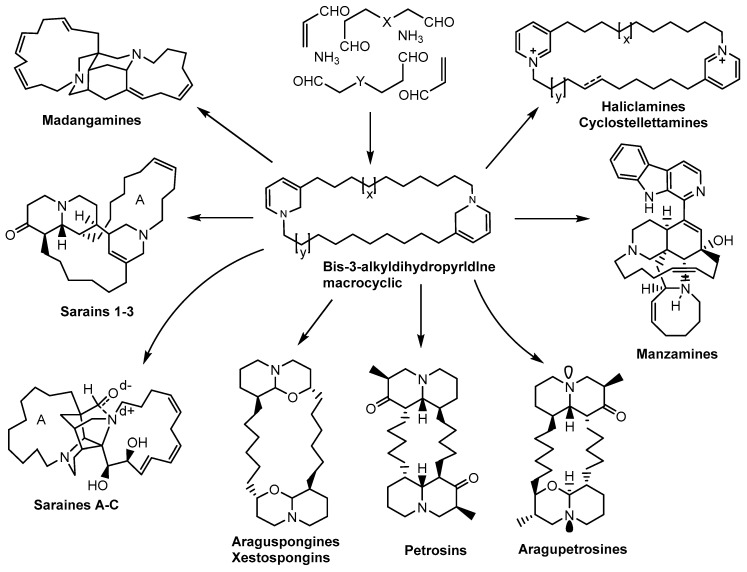
Biosynthetic considerations of the macrocyclic alkaloids originated from 3-alkylpiperidine.

Cimino et al. proposed that bis-3-alkylpiperidine was the building block of xestospongins, petrosins, and saraines [40,126]. They indicated that there was a biosynthetic relationship between the oligomeric halitoxins and the three macrocyclic alkaloids. Another study indicated a detailed hypothetical pathway for the formation of araguspongines, petrosins, and aragupetrosine A in the marine sponge *Xestospongia* sp. [19,126]. A smart study revealed the relationship between manzarnines and xestospongins, petrosins, and saraines [40]. Baldwin and Whitehead provided the first suggestion about the biogenetic origin of piperidine ring and foresaw the occurrence of ircinal A (**121**) and B (**150**) and ingenamine alkaloids (Figure 18) [39,126]. Subsequently, three studies indicated the generation of the hypothetical pathways to halicyclarnine, saraines 1–3, saraines A–C, and madangarnine skeletons [39,126]. 

The three basic building blocks of the biosynthesis of 3-alkylpiperidine alkaloids manzamine C (1**52**), keramaphidin C (**165** A), and keramamine C (**153**) include ammonia, a propenal and a variable chain of saturated or unsaturated linear dialdehyde [75,127,128].

The cross-electrophilic reaction between an equivalent of ammonia with a propenal unit and one terminus of the linear dialdehyde led to a formation of dihydropyridine, with a linear alkyl aldehyde attached at the position 3. Oxidation of the dihydropyridine ring, condensation of the free aldehyde functionality with ammonia, methoxy amine, or simple alkyl amines followed by oxidative or reductive transformations of the resulting imine led directly to monomeric 3-alkylpiperidines [75,85,129].

Chain extension occurred if the aldehyde functionality undertook reductive condensation with ammonia, another equivalent of propenal, and a terminus of another dialdehyde chain to afford a dimer with a second dihydropyridine system. Multiple replications of the elongation sequence were necessary to generate halitoxins. Cyclization involved condensation of the terminal aldehyde functionality at one end of the oligomer and the amino nitrogen in the dihydropyridine ring on the other terminus of the oligomer [129].

Cyclostellettamines result from the oxidation of the dihydropyridine rings containing appropriate linear alkyl bridges, while haliclamines result from reduction of the dihydropyridine rings. Two dialdehydes of 11 carbon atoms were required for the biogenesis of a hypothetical macrocyclic precursor of xestospongins, petrosins, araguspongines, and aragupetrosines. Oxidation of the alkyl chains to afford the diketo-macrocycle intermediate, followed by carbocyclic or heterocyclic ring formation generated either the quinolizidine or the 1-oxaquinolizidine ring systems found in the petrosins, xestospongins, araguspongines, and aragupetrosines [19]. Additionally, transformations including methylation and hydroxylation are common in the biosynthesis of petrosins, xestospongins, and araguspongines [46].

The pentacyclic skeleton of ingenamine alkaloids arose from a biological intramolecular [4 + 2] cycloaddition reaction between the tautomeric forms of the two dihydropyridine rings in a bis-3-alkyldihydropyridine macrocycle. The initial [4 + 2] adduct intermediate underwent redox exchange to obtain the pentacyclic intermediate. Hydrolysis of the iminium ion functionality led to a tetracyclic *seco* skeleton with aldehyde functionality. The skeleton and the aldehyde functionality correspond exactly to the skeleton and aldehyde functional group in ircinal A (**121**). The condensation of the ircinal-type intermediate with tryptamine and oxidation of the resulting product led to manzarnine B (**134**) (Figure 18).

Ingenamine-type intermediates were suggested as the precursors of halicyclamine A and madangamines. This can be performed through a cleavage of the C-18 and C–33 bond in the ingenarnine-type intermediate, which gives rise to the halicyclamine scaffold [129]. This biogenetic hypothesis was used to assign the relative stereochemistry at C-3 and C-l9 in halicyclamine A (**77**). Cyclization to form a quinolizidine ring system transforms a halicyclamine-type intermediate into the saraine-1 to -3 scaffold [9]. Investigation of saraine A revealed that disconnection of the C2-C3’ and C3-Nl’ bonds in saraine A (**51**) generated a halicyclamine scaffold. This confirms that the production of saraine C (**53**) from saraine A (**51**) was achieved through a halicyclamine-type intermediate [26,31,128]. Rearrangement of the ingenamine-type intermediate led to the madangamine scaffold [30,60,128]. The 3-alkylpiperidine alkaloids were isolated as racemates or unequal mixtures of enantiomers. They were produced by the same biosynthetic manifold but have opposite absolute configurations. Araguspongine B (**31**) and petrosins are reported as racemic mixtures, whereas araguspongine D (**17**) as a 3:7 mixture of (+) and (-) enantiomers, araguspongine E (**19**) as a 3:2 mixture of (+) and (-) enantiomers, and araguspongines F, G, H, J, and aragupetrosine A (**20**) as single enantiomers [5]. Araguspongines F (**33**), G (**34**), H (**35**), and J (**36**) were obtained as single enantiomers, while the related compounds were obtained as enantiomeric mixtures or *meso*-compounds; this can be explained by presuming that enantio-selective oxidation or methylation occurs at C9 or C3 prior to or after formation of intermediary 1-oxaquinolizidine moieties [46].

A comparison of the absolute configurations of manzamine A (**97**), manzamine B (**134**), ircinal A (**121**), ircinal B (**150**), ircinol A (**120**), ircinol B (**151**), ingenamine (**67**), ingamine A (**65**), ingenamine E (**71**), and keramaphidin B (**165**) indicated that all of these compounds originated from the same biosynthetic pathway of ingenamine-type intermediate [130]. **97**, **121**, 1**34**, **150**, and one enantiomer of the racemic **165** were categorized in one configuration series. Compounds **65**, **67**, **71**, **120**, **151**, and another enantiomer of **165** were categorized in another configurational series. The chirality of these alkaloids was established by the biological equivalent of an intramolecular [4 + 2] cycloaddition reaction of an achiral bis-3-alkyldihydropyridine macrocycle. Therefore, there are enzymes capable of catalyzing this intramolecular condensation [130].

## 4. Conclusion and Future Perspective

This review delivers an inclusive overview of the chemical structures and biological activities of the reported marine-derived macrocyclic alkaloids (MDMAs). There was an incredible increase in the rate of new macrocyclic alkaloids being isolated from marine-derived organisms. Up to 204 macrocyclic alkaloids have been discovered from marine organisms, particularly sponges. These metabolites were categorized under eight subclasses: pyrroles (1%), quinolines (4%), bis-quinolizidines (3%), bis-1-oxaquinolizidines (14%), 3-alkylpiperidines (25%), manzamines (34%), 3-alkyl pyridinium salts (15%), and motuporamines (4%). The majority of these metabolites were isolated from three genera, *Xestospongia*, *Acanthostrongylophora,* and *Haliclona.* MDMAs displayed potent activities that enabled them to be used as anticancer, anti-invasion, antimalarial, antiplasmodial, and antimicrobial. The reported deep-rooted mode of actions and molecular targets of these compounds were recognized. In this review, the reported structure–activity relationships (SARs) of the marine macrocyclic alkaloids, including the detailed antimalarial SAR of manzamines, were discussed. The multifunctionality of the complex chemical structures provides a wide range of different affinities to receptors. Based on the chemical diversity and biological activities of the MDMAs, it is worth studying marine sponges further to find promising lead compounds for the development of marine drugs.

## Figures and Tables

**Table 1:** List of marine-derived macrocyclic alkaloids.

Compound No.	Subclasses	Name of Compounds	Marine Organism	Biological Activities
**1–2**	Pyrroles	Densanins A and B	*Haliclona densaspicula*	Anti-inflammatory
**3–8**	Quinolines	Njaoamines A–F	*Reniera* sp.	Cytotoxic and Anti-HIV
**9–10**		Njaoamines G–H	*Neopetrosia* sp.	
**11**		Njaoamine I	*Reniera* sp.	
**12**	Bis-Quinolizidines	Petrosin	*Petrosia seriata*	
**13–14**		Petrosins A and B		
**15**		Aragupetrosine A	*Xestospongia* sp.	
**16**		Xestosin A	*Xestospongia exigua*	
**17**		Xestospongin A (Araguspongine D)		
**18**		Xestospongin B		
**19**		Xestospongin C (Araguspongine E)		
**20**		Xestospongin D (Araguspongine A)	*Xestospongia* sp.	
**21–26**		Xestospongins E–J	*Oceanapia* sp.	
**27**		(+)-7*S*-Hydroxyxestospongin A	*Xestospongia* sp.	
**28**		Demethylxestospongin B	*Xestospongia* sp. and *Neopetrosia exigua*	
**29**		3β,3ʹβ-Dimethylxestospongin C		
**30**		9′-*epi*-3β,3′β–Dimethylxestospongin C		
**31**		Araguspongine B		Antimicrobial and Cytotoxic
**32**		Araguspongine C	*Xestospongia muta*	
**33–36**		Araguspongines F–H and J	*Xestospongia* sp.	
**37**		3a-Araguspongin C	*Haliclona exigua*	
**38–39**		Araguspongines K and L	*Neopetrosia exigua*	
**40**		Araguspongine M		
**41–43**		Araguspongines N–P	*Xestospongia muta*	
**44**		*meso*-araguspongine C		
**45–47**	3-Alkyl piperidines	Saraines 1–3	*Reniera sarai*	Cytotoxic
**48–50**		Isosaraines 1–3		Antimicrobial
**51–53**		Saraines A-C		
**54–58**		Madangamines A–E	*Xestospongia ingens*	Cytotoxic
**59**		Madangamine F	*Pachychalina alcaloidifera*	
**60**		(10*E*,12*Z*)-haliclonadiamine	*Halichondria panicea*	Antimicrobial
**61**		(10*Z*,12*E*)-Haliclonadiamine	*Halichondria panacea*	
**62**		Papuamine	*Haliclona* sp.	
**63–64**		Haliclonadiamine	*Haliclona* sp.	
**65–66**		Ingamines A and B	*Xestospongia ingens*	Antimalarial
**67**		Ingenamine		
**68–72**		Ingenamines B–F		
**73**		Ingenamine G	*Pachychalina* sp.	
**74**		Dihydroingenamine D	*Petrosid Ng5 Sp5*	
**75**		22(*S*)-Hydroxyingamine A		
**76**		Xestocyclamine	*Xestospongia* sp.	protein kinase C inhibitor
**77–78**		Halicyclamines A-B	*Xestospongia* sp.	Cytotoxic
**79-80**		Haliclonacyclamines A–B	*Haliclona* sp.	
**81**		22-Hydroxyhaliclonacyclamine B	*Halichondria* sp.	
**82**		2-*epi*-Tetradehydrohaliclonacyclamine	*Halichondria* sp.	
**83**		Tetradehydrohaliclonacyclamine A mono-*N*-oxide	*Halichondria* sp.	
**84**		Tetradehydrohaliclonacyclamine A	*Halichondria* sp.	
**85**		Haliclonacyclamine C	*Haliclona* sp.	
**86**		Haliclonacyclamine D	*Haliclona* sp.	
**87**		Haliclonacyclamine E	*Arenosclera brasiliensis*	Antimalarial, Cytotoxic, Proteasome and Immunoproteasome inhibition
**88**		Haliclonacyclamine F	*P. alcaloidifera*	
**89**		Halichondramine	*Halichondria* sp.	
**90**		Neopetrosiamine A	*Neopetrosia proxima*	
**91**		Tetradehydrohalicyclamine B	*Acanthostrongylophora ingens*	
**92–94**		Arenosclerins A–C	*A. brasiliensis*	Cytotoxic, Anti-leishmanial, and Anti-HIV
**95–96**		Arenosclerins D and E	*P. alcaloidifera*	
**97**	Manzamines	Manzamine A (Keramamine A)	*Haliclona* sp.	
**98**		8-Hydroxymanzamine A (Manzamine G)	*Amphimedon* sp. and *Pachypellina* sp.	
**99**		3,4-Dihydromanzamine A	*Amphimedon* sp.	
**100**		6-Hydroxymanzamine A (Manzamine Y)	*Amphimedon* sp. and *Haliclona* sp.	
**101**		1,2,3,4-Tetrahydro-8-hydroxymanza-mine A (8-Hydroxymanzamine D)	*Cribochalina* sp. and *Petrosia* sp.	
**102**		1,2,3,4-Tetrahydro-2-*N*-methyl-8-hyd-roxymanzamine A (8-Hydroxy-2-*N*-methylmanzamine D)		
**103**		Manzamine D (1,2,3,4-Tetrahydromanzamine A)	*Ircinia* sp.	
**104**		3,4-Dihydro-6-hydroxymanzamine A	*Amphimedon* sp.	
**105**		Manzamine M		
**106**		*N*-Methyl-*epi*-manzamine D	Unidentified Paluan sponge	
**107**		*epi*-Manzamine D		
**108**		12,34-Oxamanzamine A	Sponge 011ND 35	
**109**		*ent*-8-Hydroxymanzamine A	Unidentified Indo-Pacific sponge	
**110**		12,28-Oxamanzamine A	*Acanthostrongylophora* sp.	
**111**		12,28-Oxa-8-hydroxymanzamine A		
**112**		Manzamine A *N*-oxide	*Xestospongia ashmorica*	
**113**		3,4-Dihydromanzamine A *N*-oxide		
**114–115**		Acanthomanzamines A and B	*Acanthostrongylophora* sp.	
**116**		Pre-*neo*-kauluamine	*Acanthostrongylophora ingens*	
**117**		Zamamidine C	*Amphimedon* sp.	
**118**		Zamamidine D		
**119**		Nakadomarin A		
**120**		Ircinol A		
**121**		Ircinal A	*Ircinia* sp.	
**122**		Ircinal E		
**123**		12,28-Oxaircinal A		
**124**		Manzamine E	*Xestospongia* sp.	
**125**		Manzamine F (Keramamine B)		
**126**		*ent*-Manzamine F		
**127–128**		*ent*-12,34-Oxamanzamines E and F	Sponge 011ND 35	
**129**		12,34-Oxamanzamine E	*Acanthostrongylophora* sp.	
**130**		6-Hydroxymanzamine E		
**131**		12,28-Oxamanzamine E		
**132**		12,34-Oxa-6-hydroxymanzamine E		
**133**		31-Keto-12,34-oxa-32,33-dihydroircinal A		
**134**		Manzamine B	*Haliclona* sp.	
**135–136**		Manzamines H, J	*Ircinia* sp.	
**137**		Manzamine J *N*-oxide	*Xestospongia* *ashmorica*	
**138**		8-Hydroxymanzamine B	*Acanthostrongylophora* sp.	
**139**		Manzamine L	*Amphimedon* sp.	
**140**		Manzamine B *N*-oxide	*Acanthostrongylophora* sp.	
**141**		3,4-Dihydromanzamine B *N*-oxide		
**142**		11-Hydroxymanzamine J		
**143**		Ma’eganedin A	*Amphimedon* sp.	
**144**		8-Hydroxymanzamine J	*Acanthostrongylophora*	
**145**		3,4-Dihydromanzamine J	*Amphimedon* sp.	
**146–147**		Acanthomanzamines D and E	*Acanthostrongylophora* sp.	
**148–149**		Zamamidines A and B	*Amphimedon* sp.	
**150**		Ircinal B	*Ircinia* sp.	
**151**		Ircinol B	*Amphimedon* sp.	
**152**		Manzamine C	*Haliclona* sp.	Cytotoxic
**153**		Keramamine C	*Amphimedon* sp.	
**154**		Acanthomanzamine C	*Acanthostrongylophora* sp.	
**155**		Kepulauamine A		
**156**		Acantholactam		
**157**		Acantholactone	*Acanthostrongylophora* sp.	
**158**		32,33-Dihydro-31-hydroxymanzamine A	Indonesian sponge	
**159**		32,33-Dihydro-6-hydroxymanzamine A-35-one		
**160**		32,33-Dihydro-6,31-dihydroxymanzamine A		
**161**		Manzamine X	*Xestospongia sp.*	
**162**		6-Deoxymanzamine X	*X. ashmorica*	
**163–164**		Manadomanzamines A and B	*Acanthostrongylophora* sp.	
**165**		Keramaphidin B	*Amphimedon* sp.	
**166**		Kauluamine	*Prianos* sp.	
**1** **67–1** **72**	3-Alkyl pyridinium salts	Cyclostellettamines A–F	*Stelletta maxima*	Antimicrobial and Cytotoxic
**173–1** **77**		Cyclostellettamines G–I, K, and L	*Pachychalina* sp.	
**178–179**		Dehydrocyclostellettamines D, E	*Xestospongia* sp.	
**180**		8,8‘-Dienecyclostellettamine	*Amphimedon compressa*	
**181–184**		Cyclostellettamines N, R, O, Q	*Haliclona* sp.	
**185–192**		Cyclostellettamines	*Haliclona* sp.	
**193**		Cyclostellettamine P	*Xestospongia exigua*	
**194–196**		Njaoaminiums A–C	*Reniera* sp.	Cytotoxic
**197–205**	Motuporamines	Motuporamines A–I	*Xestospongia exigua*	Anti-invasion
